# 

**Published:** 1849-11

**Authors:** 


### CONTENTS.

PART I.—ORIGINAL COMMUNICATIONS. PAGE.

ART. 1.—Cholera Infantum—By S. W. Ritchey, M.D., - - - - - - 298
ART. 2.—A Case of Scarlatina—By Wm. Matthews, M.D., - - - 306
ART. 3.—Ulcerations of the Vagina, connected with the states of Utero-Gestation and Lactation—By Daniel Brainard, M.D., - - - - - 308

PART II.—REVIEWS AND NOTICES OF NEW WORKS.

ART. 1.—Anæsthesia,, or the Employment of Chloroform in Surgery and Midwifery—By J. Y. Simpson, M.D., &c.
A Treatise on Etherization in Childbirth—By Walter Channing M.D., &c.
Effects of Chloroform and Strong Chloric Ether as Narcotic Agents—By Jno. C. Warren, M.D., &c., - - - - - - - 312
ART. 2.—Manual of Physiology—By William Senhouse Kirkes, M.D., Assisted by James Paget, Lecturer on General Anatomy and Physiology at St. Bartholomew's Hospital, - - - - - - - - 326
ART. 3.—An Introduction to Practical Chemistry, Including Analysis—By John E. Bowman, Demonstrator of Chemisty in King's College, London, - - 327
ART. 4.—On the Cryptogamous Origin of Malarious and Epidemic Fevers—By J. K. Mitchell, A.M., M.D.; Professor of Practical Medicine in the Jefferson Medical College of Philadelphia, - - - - - - 328
ART. 5.—A Manual of Auscultation and Percussion—By M. Barth, Agrege to the Faculty of Medicine of Paris, &c., and M. Henry Roger, Physician to the *Bureau Centrale* of the Parisian Hospitals, - - - - - 339
ART. 6.—Clinical Midwifery. Comprising the Histories of Five Hundred and Forty-five Cases of Difficult, and Preternatural Labor. With Commentaries: By Robt. Lee, M.D., F.R.S., - - - - - - 342
ART. 7.—The Practice of Surgery: Embracing Minor Surgery, and the Application of Dressings, etc., etc.—By John Hastings, M.D., U.S.N., - - 343
ART. 8,—Chemical Analysis, Qualitative and Quantitative—By Henry M. Noad, Lecturer on Chemistry at St. George's Hospital, &c., - - - - 344
ART. 9.—Human Anatomy—By Jones Quain, M.D., Edited by Richard Quain, F.R.S., and William Sharpey, M.D., Professors of Anatomy in University College, London, - - - - - - - - - 345
ART. 10.—Report on the Practical Operation of the Law Relating to the Importation of Adulterated and Spurious Drugs, &c.—By M. J. Bailey, M.D., - 349

PART III.—SELECTIONS.

ART. 1.—Infantile Paroxysmal Convulsions, and their Treatment with Sulphate of Quinine, with Cases—By Henry F. Campbell, M.D. - - - - 352
ART. 2.—Homœopathic Trumpet—An Uncertain Sound, - - - - 356
ART. 3.—Nux Vomica in Intestinal Obstructions, - - - - - 362
ART. 4.—Free Medical Schools. - - - - - - - 362

PART IV.—EDITORIAL.

ART. 1.—Medical Societies, - - - - - - - 364
ART. 2.—Free Medical Schools, - - - - - - - 365
ART. 3.—Obituary, - - - - - - - - - 370
ART. 4.—Miscellaneous Intelligence, - - - - - - 370

